# Autism-associated miR-873 regulates *ARID1B, SHANK3* and *NRXN2* involved in neurodevelopment

**DOI:** 10.1038/s41398-020-01106-8

**Published:** 2020-12-01

**Authors:** Jing Lu, Yan Zhu, Sarah Williams, Michelle Watts, Mary A. Tonta, Harold A. Coleman, Helena C. Parkington, Charles Claudianos

**Affiliations:** 1 Monash Biomedicine Discovery Institute, Monash University, Melbourne, VIC 3800 Australia; 2 Monash Bioinformatics Platform, Monash University, Melbourne, VIC 3800 Australia; 3 Queensland Brain Institute, The University of Queensland, Brisbane, QLD 4072 Australia; 4 Centre for Mental Health Research, The Australian National University, Canberra, ACT 0200 Australia

**Keywords:** Clinical genetics, Molecular neuroscience

## Abstract

Autism spectrum disorders (ASD) are highly heritable neurodevelopmental disorders with significant genetic heterogeneity. Noncoding microRNAs (miRNAs) are recognised as playing key roles in development of ASD albeit the function of these regulatory genes remains unclear. We previously conducted whole-exome sequencing of Australian families with ASD and identified four novel single nucleotide variations in mature miRNA sequences. A pull-down transcriptome analysis using transfected SH-SY5Y cells proposed a mechanistic model to examine changes in binding affinity associated with a unique mutation found in the conserved ‘seed’ region of miR-873-5p (rs777143952: T > A). Results suggested several ASD-risk genes were differentially targeted by wild-type and mutant miR-873 variants. In the current study, a dual-luciferase reporter assay confirmed miR-873 variants have a 20-30% inhibition/dysregulation effect on candidate autism risk genes *ARID1B, SHANK3* and *NRXN2* and also confirmed the affected expression with qPCR. In vitro mouse hippocampal neurons transfected with mutant miR-873 showed less morphological complexity and enhanced sodium currents and excitatory neurotransmission compared to cells transfected with wild-type miR-873. A second in vitro study showed CRISPR/Cas9 miR-873 disrupted SH-SY5Y neuroblastoma cells acquired a neuronal-like morphology and increased expression of ASD important genes *ARID1B*, *SHANK3*, *ADNP2, ANK2* and *CHD8*. These results represent the first functional evidence that miR-873 regulates key neural genes involved in development and cell differentiation.

## Introduction

Autism spectrum disorders (ASD) are characterised by impaired social interaction, communication deficits, restricted and repetitive behaviour, and affect 1–2% of the world population. Extensive research has been conducted to identify the genetic causes of ASD with literally hundreds of genes now linked to ASD risk^1^. Currently, there are 913 genes represented in the Human Gene Module from Simons Foundation Autism Research Initiative (SFARI), a comprehensive resource on ASD genetics^2^. Although there are few common mutations of high frequency, many rare variations occur in risk genes associated with neurodevelopment and synapse development^3^. Among them, *SHANK3* encodes a notable protein scaffolding molecule that has been well studied in animal models; genetic disruption of which, results in autistic-like behaviours and neuronal morphologic deficits^4^.

Although significant efforts have been made to examine the role of DNA variations found in protein coding genes, there remains a paucity of knowledge concerning the large number of variations detected in noncoding DNA including microRNAs (miRNAs) and their target sites in mRNAs^5^. MiRNAs are short (~22 nucleotides) noncoding RNAs that regulate gene expression at all known levels of cell development, including the nervous system^6–9^, and are thought to play a vital role in autism^10–12^. Unfortunately, there is little direct functional evidence to support this assumption. The relationship between gene expression and large copy number variations (CNVs) containing miRNAs has also been linked with ASD. A recent study showed that miR-137 targets 3’-untranslated region (3’UTR) of the candidate autism gene *RORa*^13^, but there has yet to be a definitive association of DNA variation of any specific miRNA with ASD^14^.

Current evidence suggests miRNAs usually bind to the 3’UTR of the target mRNAs to regulate protein synthesis^15^. The first 2–8 nucleotides are generally required for miRNA binding and thus termed as a ‘seed region’; however, a single nucleotide variation (SNV) in a mature miRNA sequence may also change the stability of its precursor miRNA hairpin, or alter the mature miRNA targeting specificity^15^. We previously conducted high-efficiency whole-exome sequencing of Australian families with ASD (*N* = 128; 48 ASD cases and 80 parents)^5,16^ and recently reported a landmark noncoding DNA sequence analysis associated with this autism cohort. Among the many sequence variations found to occur in regulatory DNA regions of the genome, we identified four rare SNVs within mature miRNA sequences. Of interest was an inherited SNV (rs777143952; T > A) of miR-873-5p (gene ID: 100126316) found in an idiopathic ASD case. This individual inherited this miR-873 mutation from an unaffected father together with a putative disruptive *NRXN1* coding mutation (chr2:50847195; G > A; NM_004801: exon8:c.C1285T:p.P429S) from a mother diagnosed with broader autism phenotype (BAP)^5,16^. *NRXN1* is a notable autism risk gene, deletions and disruptions of which have been reported in several ASD cases^17–19^. MiR-873 is located within an intron of the neural gene *LINGO2*, which is highly expressed in the brain^20^, not surprisingly, miR-873 is also highly expressed in many brain regions^21^. For this miR-873 mutation, the maximum population allele frequency is 0.000007130^22^. MiR-873 is very conserved and it was annotated on 17 species in miRbase^23^. Interestingly, miR-873 is also found to be downregulated in brains with glioblastoma tumours^24^ and with learning difficulties linked to a chromosomal microdeletion that includes miR-873^25^. As previously stated^16^, we propose that both coding (e.g. *NRXN1*) and noncoding miR-873-5p variants synergise to increase genetic liability in this ASD case.

Our previous analyses suggested several key ASD-risk genes are likely targets of miR-873 variants^16^. We now confirm several risk genes including *ARID1B* and *SHANK3* are regulated by miR-873. Moreover, changes in expression of miR-873 are shown to affect the morphology of neurons and differentiation of SH-SY5Y cells.

## Materials and methods

### MicroRNA probes

MicroRNA mimics are chemically modified double-stranded RNAs that mimic endogenous miRNAs. The following microRNA mimics were purchased and employed in this study: wild-type (WT) miRNA-873-5p mimics (5’-GCAGGAACUUGUGAGUCUCCU), mutant (Mut) miRNA-873-5p mimics (5’-GCUGGAACUUGUGAGUCUCCU), control miRNA mimics cel-miR-239b from *C. elegans*, targeting no human genes (5’- UUUGUACUACACAAAAGUACUG), and an inhibitor, antisense RNA of miR-873-5p (5’-AGGAGACUCACAAGUUCCUGC) (Dharmacon, Chicago, IL, USA).

### Bioinformatic analyses

The targeting genes of WT and Mut miR-873 were pulled down using biotin-conjugated miRNA mimics in SH-SY5Y and the sequencing data have been submitted to GenBank under the Accession number GSE98088^16^. Differential gene enrichment between WT and Mut miR-873, and respective controls was calculated with DeSeq2^26^ as previously described. *P* values were corrected with the Benjamini–Hocheberg algorithm. Genes were considered as differential targeting if the adjusted *P* value was <0.01 and absolute fold-enrichment (FE) was ≥2. Gene Ontology (GO) term analysis of differentially enriched genes of WT and Mut miR-873 (*P* < 0.01, FE ≥ 2) was performed using the Protein ANalysis THrough Evolutionary Relationships (PANTHER) Classification System, version 13.0^27^. The biological process GO_BP term of WT and Mut miR-873 were further clustered with REVIGO^28^. The top ten terms were subsequently plotted in GraphPad Prism and relationship of GO_BP terms were visualised using Cytoscape. Pathway and network analyses were conducted with Ingenuity Pathway Analysis (IPA, Qiagen). The list of differentially targeting genes of WT and Mut miR-873 containing gene IDs, fold changes and *P* values were uploaded into the IPA tool. The WT or Mut miR873 specific targeting genes associated with human diseases were analysed and filtered with “neural disease”. Then six autism related diseases: aphasia, speech disorders, cognitive impairment, bipolar disorder, autism and mental retardation were diagrammed in IPA. For the miRNA shared target analysis, the WT miR-873 target genes were uploaded to the IPA software. Then a list containing miR-124, miR-125 and miR-663 was created. The shared gene targets between miR-873 and other miRNAs were compared. To identify the roles of those differentially targeted genes in ASD, we curated critical genes in autism databases SFARI (Simons Foundation)^2^ and Autism KB (the Centre for Bioinformatics, PKU)^29^.

### MiRNA-873 gene targets validation using the dual-luciferase reporter system

The methodological details for this experiment are available in two recent publications from our laboratory^30,31^. The dual-luciferase reporter assay (ΨCheck-2 Promega^®^) was used to confirm interactions between miR-873 and miRNA regulatory elements (MREs) of candidate genes in the monkey kidney Cos-7 cell system. First, MREs were predicted with MIRANDA^32^, then ~200–300 bp gene sequence containing MRE was cloned into ΨCheck-2 vector before *Renilla* luciferase (pirmers were listed in Supplementary Table 1). The expression of *Renilla* luciferase is regulated by the sequences cloned in. The ΨCheck-2 vector also carries a firefly luciferase to monitor the expression levels of the vector in different cells. The constructed vector 1 µg and miRNA mimics 20 pmol were transfected into Cos-7 cells with Lipofectamine 2000 (Thermo Fischer Scientific, Waltham, MA, USA), the luciferases were allowed to express for 24 h. Then samples were prepared according to the manufacturer’s instructions (Promega, Madison, WI, USA). Luciferase activity was measured using a CLARIOstar microplate reader (BMG LABTECH, Germany). The *Renilla*/firefly ratio was calculated, and the percentage of the downregulation was normalised to control cel-miR-293b transfected group. Four independent experiments were conducted.

### Gene expression levels measured by qPCR

Gene expression levels were measured with quantitative PCR (qPCR), with the methods reported previously^33^. The cells were transfected with 20 pmol each for WT, Mut miR-873 and cel-miR-293b control with Lipofectamine 2000. RNA was extracted with TRIzol reagent (Thermo Fisher Scientific) and reverse transcribed with SuperScript™ IV First-Strand Synthesis System (Thermo Fisher Scientific), then PCR was performed with QuantiFast SYBR^®^ Green PCR Kit (Qiagen) on a LightCycler^®^ 480 Instrument (Roche, Switzerland). The expression levels of miR-873 was quantified with the stem-loop RT-PCR method^34^. GAPDH was used as a control for coding gene and U6 snRNA were used as a control for quantifying miRNA. Four replicates were carried out in each group. All primers were listed in Supplementary Table 1.

### Primary neuron culture, transfection, imaging and electrophysiological recording

Primary hippocampal neurons from wild-type (E18) C57BL/6J mice were cultured according to the published protocols^35^. All the procedures were approved by Monash University Animal Ethics Committee MARP-2 under the approval number MARP/2015/124. The neurons were seeded in a 24-well plate with poly-D-lysine coated glass coverslips at 5 × 10^4^ cells per well. At 11 days in vitro (DIV) culture, the neurons were transfected with 20 pmol each WT, Mut or inhibitors of miR-873 and cel-miR-239b control using the Lipofectamine^®^ LTX (Thermo Fischer Scientific). After 72 h, the neurons were immunostained with dendritic marker Map2 (Abcam, ab5392) and images were taken using Zeiss, AXIO Imager M2 with ZEN software (version 2.0, Germany). Morphology changes (branch number and dendrite length) were graphically and statistically analysed with Image J plugin Sholl analysis^36^. The number of intersections over the distance from the cell soma, was plotted as a curve for each of the four groups to visualise the average distribution of branches and length of dendrites among four groups. There are more than 60 neurons quantified from each group.

For electrophysiological characterisation of the neurons, coverslips containing transfected neurons were transferred to a recording bath (Warner Instruments, Hamden, CT, USA) and were continuously superfused with physiological saline solution (PSS) containing (mM): NaCl 137, NaHCO_3_ 4, NaH_2_PO_4_ 0.3, KCl 5.4, KH_2_PO_4_ 0.44, MgCl_2_ 0.5, MgSO_4_ 0.4, glucose 5.6, HEPES 10, CaCl_2_ 1.5 at pH 7.4 at room temperature. Glass electrodes (PG150T-15, Harvard Apparatus, Kent, UK) were pulled (P-97, Sutter Instruments, CA, USA) and fire polished (MG-80, Microforge, Narashige, Japan). The electrodes were filled with solution containing (mM): KCl 10, CaCl_2_ 0.05, Mg_2_ATP 4, K-gluconate 130, Na_2_-phosphocreatine 10, EDTA 0.01, EGTA 0.1, HEPES 10, pH 7.2 and had resistances of 2–5 MΩ. Electrophysiological activity was recorded using the patch-clamp technique in whole-cell mode using an Axopatch 200 A series amplifier (Axon Instruments, CA) controlled by pCLAMP v.10 software (Molecular Devices, San Jose, CA, USA). Data were digitised at 5–20 kHz and analysed using Clampfit 10 (Molecular Devices). Current clamp mode was used to record the resting membrane potential, input capacitance and resistance. Action potentials were evoked using depolarising current steps. Voltage-clamp mode using 10 mV depolarising steps in 10 mV increments, from a holding potential of –100 mV, was used to record voltage-gated inward currents. Tetrodotoxin (TTX) was added to the PSS to block Na^+^ currents (Sigma–Aldrich, St. Louis, MO, USA). Seven neurons in each group were recorded. The preparations were fixed in paraformaldehyde 4% immediately upon termination of electrophysiology and were later stained immunohistochemically.

### CRISPR/Cas9 knockout of miR-873 in SH-SY5Y cell

Plasmid pSpCas9(BB)-2A-Puro (PX459) was requested from Dr. Feng Zhang’s lab at Broad Institute through Addgene (#62988) and then constructed with protospacer sequences of CRISPR/cas9 against miR-873. The single guide RNA (gRNA) was designed by CRISPR DESIGN (http://crispr.mit.edu/)^37^. The primers for cloning the gRNA sequence were synthesised by Integrated DNA Technologies (Coralville, IA, USA), and then annealed and cloned into PX459 plasmid. The validated plasmid containing the gRNA sequence and Cas9 was transfected to SH-SY5Y cells with Lipofectamine 2000, and cells were selected with 2 µg/ml puromycin for 6 days. Then single cell was plated into a 96-well plate for proliferation. Cells presentated with two different morphologies, the cells with neuronal-like long processes were maintained and Sanger sequencing confirmed. Five potential CRISPR off target genes were confirmed through Sanger sequencing. Anti-βIII tubulin antibody (Abcam, ab18207) was used to indicate the neuron-like feature in those cells with or without miR-873 transfection. The morphology of neurons was further analysed with Sholl analysis, more than 30 neurons were quantified from each group. All primers were listed in Supplementary Table 1.

### Statistical analysis

Where required *P* values were adjusted using Benjamini–Hochberg correction for bioinformatics analysis. One-way ANOVA was used for group comparison, and the *P* values for comparison with control groups were obtained from one-way ANOVA; the *P* values for comparison with the effect of WT and Mut miR-873, one-tailed or two-tailed Student’s *t*-test was employed. Other statistical analysis was conducted as indicated in the figure legends.

## Results

### Identification of mRNA targets of WT and Mut miRNA-873

**Fig. 1:**
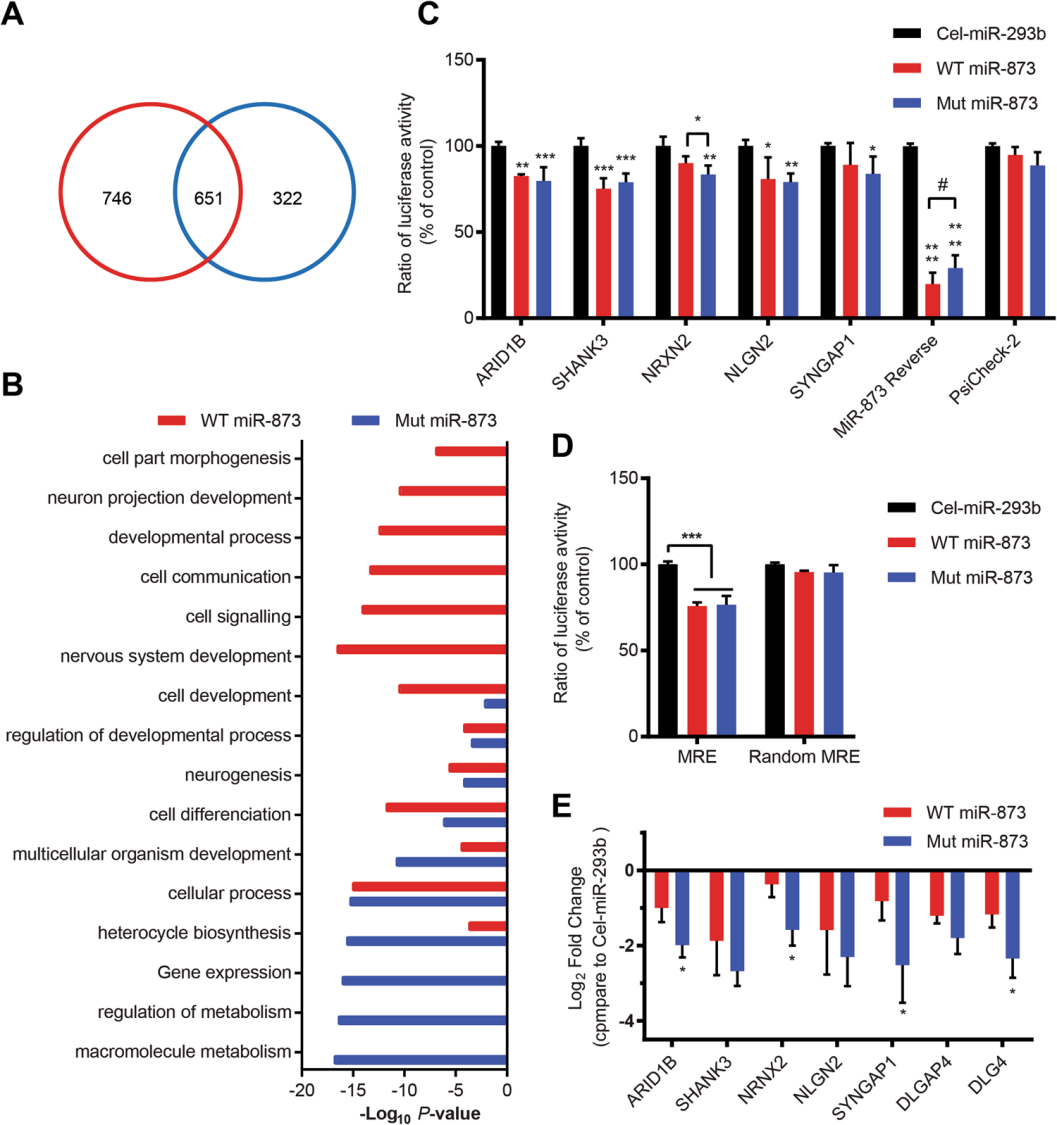
Bioinformatic analysis of pulled-down mRNAs by WT and Mut miR-873 and validation with DLA and qPCR. **a** Number of significant pulled-down mRNAs compared with control miRNA cel-miR-67. WT (746 + 651, red) and Mut miR-873 (322 + 651, blue) have 651 gene targets in overlap. **b** Top ten GO_BP terms with WT and Mut miR-873 pulled-down genes. **c** Decrease in expression ratio of *Renilla* luciferase containing putative MRE sites of target genes in Cos-7 cells cotransfected with WT, Mut miR-873 and cel-miR-239b. Empty ΨCheck-2 vector was used as a negative control, and the vector containing complete reverse sequence of miR-873 was used as a positive control. **d** Decrease in expression ratio of *Renilla* luciferase containing putative MRE-1 of *SHANK3* and a random MRE in Cos-7 cells cotransfected with WT, Mut miR-873 and cel-miR-239b. Data are presented as mean ± SD. *N* = 4. **e** QPCR showing gene expression levels after transfection with WT and Mut miR-873, control cel-miR-293b. Data are presented as mean ± SD. *P < 0.05, **P < 0.01, ***P < 0.001, ****P < 0.0001. *N* = 4.

Our previously reported pull-down transcriptome analysis used biotin-conjugated miRNA mimics to identify bound mRNA targets in human neuroblastoma SH-SY5Y cells and results were deposited in GenBank (accession number GSE98088)^16^. In contrast with our previous analysis which used *P* < 0.05^16^, we now use a more rigorous threshold (*P* < 0.01) for choice of candidate target genes of miR-873 for functional analysis. We identified a total of 1719 genes of which 746 are possible unique targets of WT and 322 are unique targets of Mut miR-873, including 651 genes in common [fold enrichment (FE) > 2], indicating a substantial change in the targeting profile (Fig. 1a, Supplementary Fig. 1 and Supplementary Data 1). Gene Ontology_Biological Process (GO_BP) terms were analysed, and the interaction network was created through Cytoscape (Supplementary Data 2). Interestingly, the connections formed within WT miR-873 GO_BP terms are considerably more complex than those formed by Mut miR-873 GO_BP terms (Supplementary Fig. 2). The top ten terms of WT targets are more related with neuronal functions whereas the Mut targets show a relatively nonspecific pattern of biological functions (Fig. 1b) suggesting Mut miR-873 may have lost functions related to binding and regulating genes involved in neurodevelopment including those involved in autism, speech disorders and mental retardation (Table 1).

**Table 1:** MiR-873-associated neuronal diseases and function pathways.

	WT miR-873						Mut miR-873	
Disease	Autism	Aphasia	Speech and language disorders	Cognitive impairment	Mental retardation	Bipolar disorder	Mental retardation	Bipolar disorder
P value	1.82E-02	3.39E-03	5.49E-03	1.67E-02	1.84E-02	1.71E-02	4.15E-03	1.25E-02
Genes	*ACE, HTR6, ACHE, DRD4, GRIN1, MECP2, TCF7L2, GRIN2D*	*GRN, ACHE, DRD4, GRIN1, GRIN2D*	*GRN, HTR6, DRD4, ACHE, GRIN1, TCF7L2, GRIN2D, CHAMP1*	*ACE,ACHE,ADAT3,ATP1A3, CASP2,CHAMP1,CNNM2, DCX,DRD4,EEF1A2,FASN, GABBR1,GATAD2B,GDI1, LARGE1,GRIN1,GRIN2D, MBOAT7,MECP2,GPC1, TECR,TRAPPC9,TSPAN7, MED25,PYCR1,TCF7L2, HTR6,INPP5E,KANSL1*,	*ADAT3,CASP2,CHAMP1, CNNM2,DCX, MBOAT7, EEF1A2,FASN,GABBR1, MED25,PYCR1,TCF7L2, KANSL1,LARGE1,TECR, GATAD2B,GDI1,GPC1, GRIN1,HTR6,INPP5E, TRAPPC9,TSPAN7, DRD4, MECP2*,	*CDC25B, WHRN, NDUFS8,OGG1, GRIN1,GRIN2D, PSKH1,PTPRU, ACHE,ATP1A3, KCNH2,NCS1, DEGS2,DRD4, ABLIM1,ACE, GRM4,HTR6, CHRNB2*	*CHRM1,CREBBP, SETBP1,T3GAL3, BCL11A,MED12, SIGMAR1,EDC3, ARID1B,EP300, ARID1A,LATS1, SMARCB1, AUTS2*	*SYN2, SYN3, THRA, EYA3, KCNN3, CHRM1, PRKCA, ZNF490, SLC18A1, SIGMAR1*,

Among the common targets 37 were associated with the SFARI geneset and 119 with the AutismKB database^29^, all of which were recognised autism risk genes (Supplementary Fig. 3). Among nonshared target genes, we found 42 (WT-targeted) vs 22 (Mut-targeted) in the SFARI set and WT 119 vs Mut 56 targets in AutismKB (Supplementary Fig. 3), respectively. Notable among these were the chromatin remodelling gene *ARID1B* and synaptic genes *SHANK3* and *NRXN2*. These results indicate one SNV alteration greatly changed the targeting profile and functions of miR-873.

### MiR-873 target gene validation using dual-luciferase reporter system

To test whether targeting interactions of miR-873 promote mRNA degradation, we employed a dual-luciferase reporter assay with the Cos-7 cell system as previously described^31,33,38^. MIRANDA predicted miRNA-873 recognition elements (MREs) were cloned into ΨCheck-2 vector before Renilla luciferase. Targeted *Renilla* luciferase expression was measured against constitutively expressed Firefly luciferase, which was also used as an internal control to monitor transfection efficiency. The empty vector was used as an negative control, and the vector containing miR-873 reverse sequence was used as a positive control. The positive control vector constructed using perfectly matched target sites for miR-873 showed the strongest suppression with 80.0 ± 2.1% decrease in expression (luminosity) of chimeric *Renilla* luciferase in the presence of the WT miR-873 mimic (Fig. 1c), while the suppression effect was decreased to 70.7 ± 10.2% with Mut miR-873 confirming a little reduced binding affinity (*P* = 0.056). For cells containing vectors with cloned MREs of *ARID1B*, *SHANK3*, *NRXN2*, *NLGN2* and *SYNGAP1*, WT and Mut miR-873 exerted similar suppression effect on expression of *Renilla* luciferase with decreases of 17.4 ± 6.5 vs 20.4 ± 9.3%; 24.8 ± 13.8 vs 21.1 ± 10.1%; 10.0 ± 1.0 vs 16.5 ± 5.5%; 19.1 ± 8.2 vs 20.9 ± 10.0%; 10.9 ± 10.1 vs 16.1 ± 5.1%, respectively (Fig. 1c). The targeting is significantly different between WT and Mut miR-873 to *NRXN2* with one-tailed Student’s *t*-test. To confirm the dysregulation specifically dependent on the MRE within cloned sequences, we compared MRE-1 of *SHANK3* and a randomised MRE sequence (Supplementary Table 2). Results confirmed an inhibition effect for MRE-1 but not for the random sequence for both WT and Mut miR-873 (Fig. 1d). The above results confirmed that miR-873 variants specifically target candidate MRE sequences from those autism risk genes. A ~20% reduction in expression of chimeric *Renilla* luciferase proteins are consistent with levels reported from previous studies using this system^31,33,38^.

Pull-down RNA sequencing identified differential targeting of WT and Mut miR-873 and the binding and repressing effect of key gene targets were validated using dual-luciferase assay and expression level of ASD-risk genes measured using quantitative PCR (qPCR) (Fig. 1e). Besides WT and Mut miR-873, control cel-miR-293b mimics with no-known human targets were compared. After transfection with mimics, the mRNA levels of the autism risk genes (i.e. *ARID1B*, *SHANK3*, *SYNGAP1*) and synaptic genes (i.e. *NRXN2, DLGAP4*, *DLG4*, *NLGN2*) were found to be downregulated by 2-4 fold in WT and Mut miR-873 compared to cel-miR-293b control. Interestingly, *ARID1B*, *NRXN2*, *SYNGAP1* and *DLG4* were significantly downregulated by Mut miR-873 (Fig. 1e), suggesting a gain-of-function rather than a loss-of-function. Although results confirmed an inhibition effect of both WT and Mut miRNA-873, Mut miRNA-873 did show a measurable difference in binding affinity to some mRNAs compared to WT and consequently changes in gene expression of those targets. These results more generally indicate miR-873 targets autism risk including key synaptic genes, involved in neurodevelopment and neural function.

### MiR-873 effects on neuronal development of mouse primary hippocampal neurons

Mature miR-873-5p is identical in human and mouse. Two MREs of miR-873 in *SHANK3* were identified as being near identical between human and mouse. Their free energy binding score are −25.41 and −19.15 kCal/mol, respectively (Supplementary Table 2). Targeted dysregulation as implied by dual-luciferase assay (Fig. 1d) suggests miR-873 will regulate human and mouse *SHANK3* transcripts in a similar mode.

**Fig. 2:**
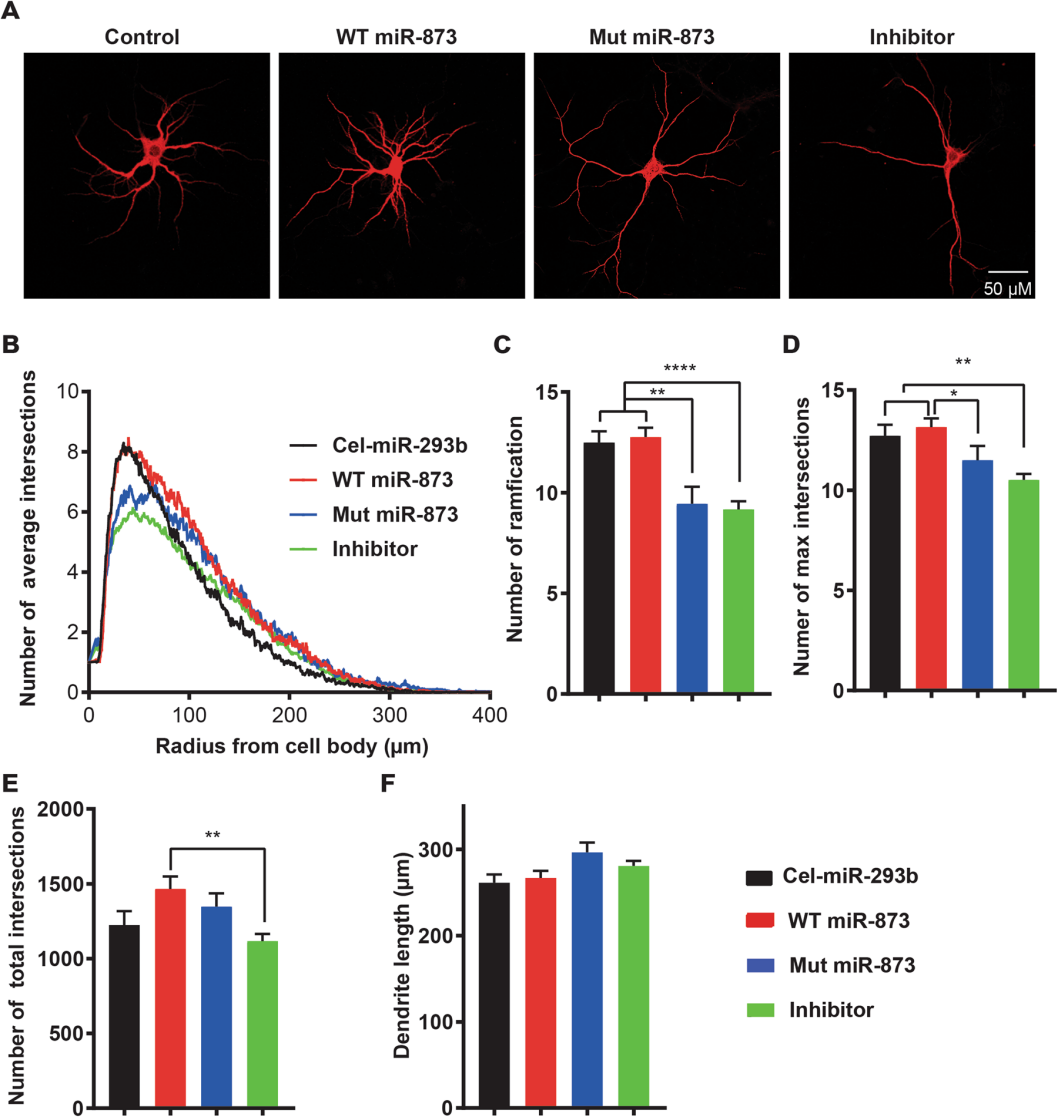
Morphological changes of primary neurons after transfection with WT and Mut miR-873. **a** Representative images of mouse neurons after transfection with WT, Mut, inhibitor miR-873 and cel-miR-293b. Bar = 50 µm. **b** Number of average intersections on the above four groups analysed with Sholl analysis. **c**–**f** Number of ramification (**c**), number of max intersections (**d**), number of total intersections (**e**), and dendrite length (**f**), analysed with Sholl analysis. One-way ANOVA was used to compare the WT, Mut miR-873 to the cel-miR-293b group. Data are presented as mean ± SEM. *P < 0.05, **P < 0.01, ****P < 0.0001. *N* = 60.

To examine the role of miR-873 on neuronal development, we assessed the neuronal morphologies using Sholl analysis^36^ after treansfection with WT, Mut and inhibitor of miR-873 and cel-miR-293b (Fig. 2a). Neurite outgrowth and dendritic branching are critical for the formation and maintenance of the neuronal circuits. The average number of intersections in each group over the distance from the cell soma was calculated and plotted as a curve (Fig. 2b). The number of ramification of neurons in control and WT miR-873 transfected groups was found to be significantly higher than those transfected with miR-873 inhibitor (12.5 ± 4.1, 12.8 ± 4.2 vs 9.4 ± 4.8, 9.2 ± 4.2) showing a 25% increase in neuronal complexity/arborisation (Fig. 2a–c). Notably, the maximum number of intersections in control and WT treated groups was higher than that in both Mut and inhibitor treated groups (Fig. 2b, d). The total intersections being significantly higher in the WT compared to inhibitor group (Fig. 2e). The length of the neuron branches does not show a discernible difference between groups (Fig. 2f). Overall, WT miR-873 was shown to maintain an increased cell complexity with more elaborate arborisation of neurons compared to Mut and inhibitor miR-873 treatments. This in vitro study suggests WT miR-873 has gene regulatory functions associated with neurodevelopment and the Mut miR-873 variant is functionally compromised.

### MiR-873 effects on electrophysiological characteristics of primary hippocampal neurons

**Fig. 3:**
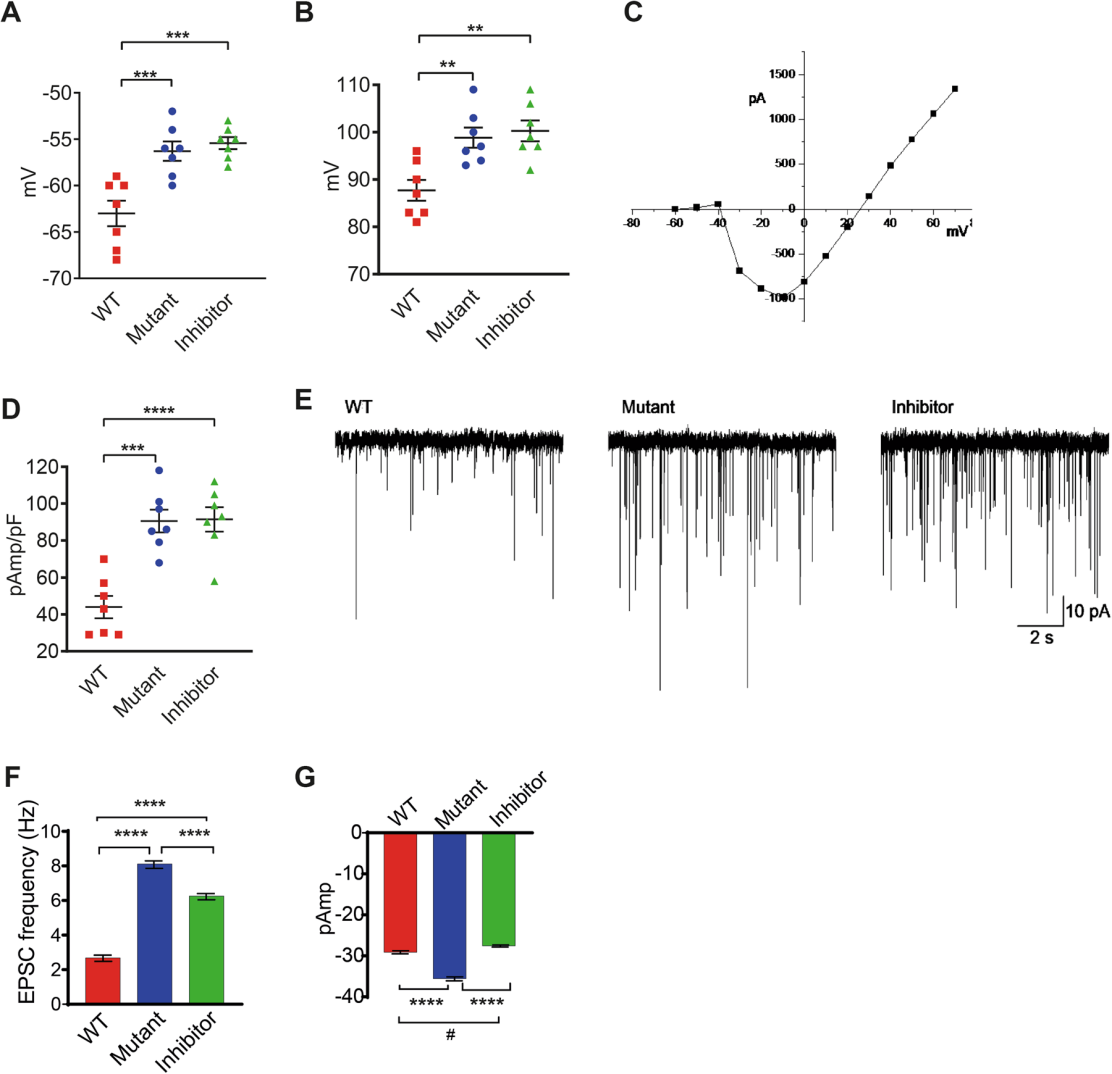
Alteration of characteristic and synaptic function of primary neurons after transfection with WT and Mut miR-873. Patch-clamp electrophysiology in current clamp mode showed **a** membrane depolarization and **b** action potential amplitude in mouse primary hippocampal neurons. **c** Sodium currents were recorded in voltage-clamp mode. **d** Changes in the maximal amplitudes of the voltage-gated sodium current were shown. **e** Excitatory postsynaptic currents (EPSCs) occurred spontaneously in all treatment groups. **f** Changes in frequency and **g** amplitude of EPSCs in neurons followed transfection with WT, Mut, and inhibitor miR-873.

In current clamp mode, resting membrane potentials of hippocampal neurons transfected with Mut or inhibitor miR-873 were more depolarised than neurons transfected with WT miR-873 (Fig. 3a). The amplitude of the action potential (AP) (Fig. 3b) was significantly larger in mutant miR-873 and inhibitor transfected neurons and this was supported by an enhanced underlying sodium current (Fig. 3c, d). Excitatory postsynaptic potentials (EPSPs) reached threshold for the firing of APs and hence the underlying currents, EPSCs, were studied in the presence of tetrodotoxin (TTX 100 nM) to block APs. EPSCs occurred more frequently in mutant miR-873 and inhibitor transfected neurons (Fig. 3e, f). However, EPSC amplitude was enhanced only in mutant miR-873 neurons (Fig. 3e, g). The frequency of EPSCs was not influenced by blockade of inhibitory neurons with picrotoxin (30 µM), but they were abolished by CNQX (100 µM), which blocked AMPA receptors. The results confirm neuronal signalling is changed in mutant miR-873.

### CRISPR/Cas9 knockout of miR-873 in SH-SY5Y cell promoting cell differentiation

**Fig. 4:**
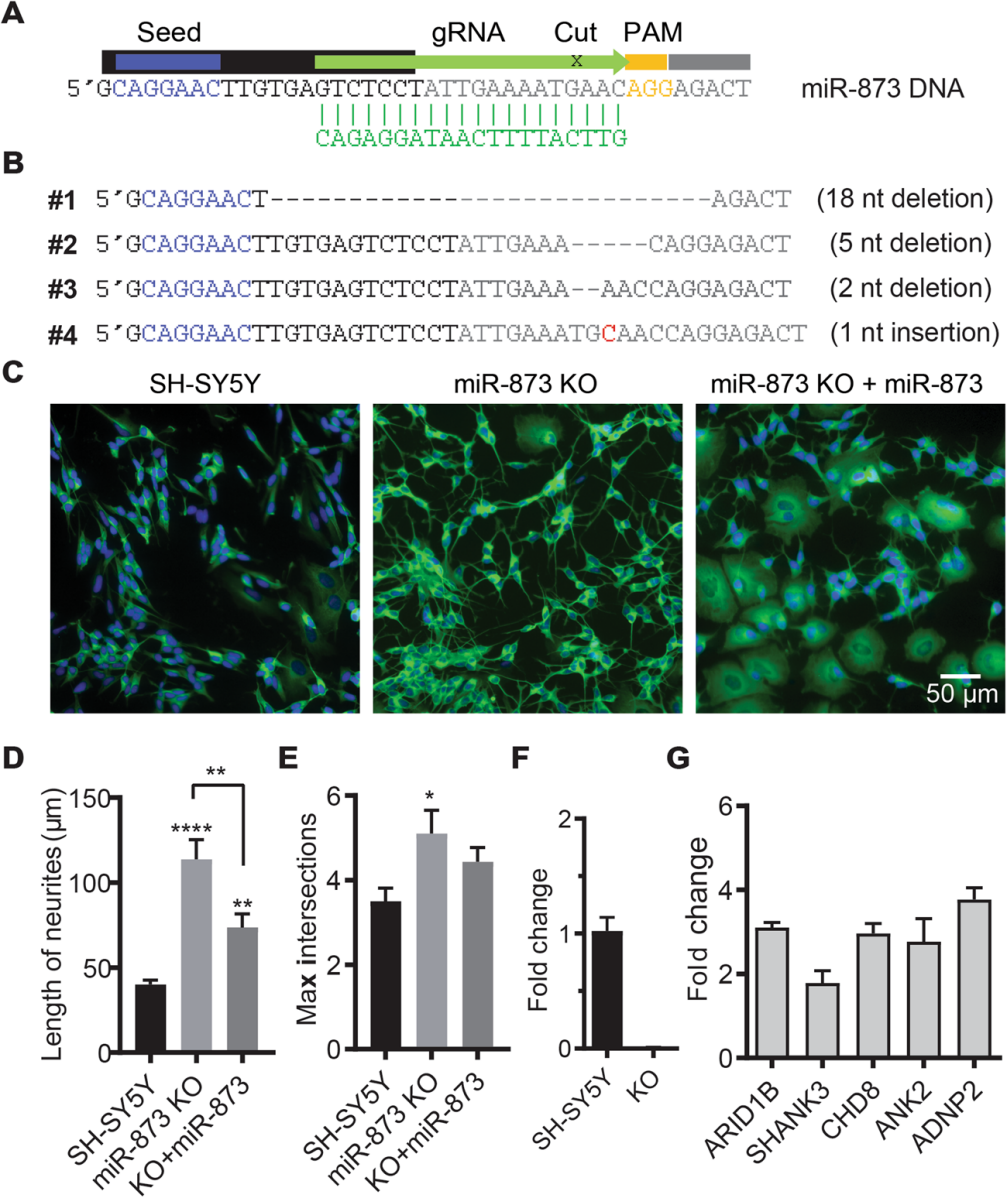
CRISPR/Cas9 MiR-873 knockout in SH-SY5Y cells. **a** Design of guide RNA (gRNA) used for the CRISPR/Cas9 genome editing. PAM sequence, orange; gRNA, green; miR-873 mature sequence, black; seed, blue. **b** CRISPR/Cas9 generated indels in four miR-873 gene disrupted cell clones. **c** βIII tubulin staining of 6-day cultured control (SH-SY5Y) cells, miR-873 KO (clone #1) and WT miR-873 mimics transfected cells (clone #1 + WT miR-873). **d** Neurite length and number of max intersections (**e**) were analysed with Sholl analysis. Data are presented as mean ± SEM. *P < 0.05, **P < 0.01, ****P < 0.0001. *N* = 30. **f** QPCR confirmed loss of miR-873 expression in KO cells. U6 snRNA was used as an internal control. *N* = 3. **g** Expression levels of candidate ASD genes in miR-873 KO cells. GAPDH was used as an internal control. Data are presented as mean ± SD. *N* = 3.

To identify roles of miR-873 in cell development, miR-873 was disrupted using CRISPR/Cas9 system, the experimental strategy is shown in Fig. 4a. There were two different cell morphologies after single cell screening, and the ones with neuronal-like longer branches were found to contain miR-873 mutations (Supplementary Fig. 4). Four cell clones were subsequently sequenced and characterised as having various insertion and deletions (INDELs) that possibly disrupted miR-873 function (Fig. 4b). Sangering sequencing data suggested, clone #1, #3, #4 were potentially result from a biallelic cut, as exihited a single sequence, while clone #2 was potentially a single allelic mutation with multiple sequences. Clone #1, containing an 18 nt deletion with 12 nt overlapped with miR-873 sequence, was chosen for further analysis. SH-SY5Y cells (control) and miR-873 KO (clone #1) cells were compared. Remarkably, we observed miR-873 KO SH-SY5Y cells differentiate into a neuronal morphology with marked increase of neurite-like outgrowth without application of transformation factors (e.g. retinoic acid). This neuron-like morphology and differentiation could be supressed/reversed when cells were transfected with miR-873 for 3 days as shown by βIII tubulin stained cells (Fig. 4c). After miR-873 knockout in SH-SY5Y cell, the neurite length had increased from ~45 to 123 µm, and the number of branches increased from ~3.8 to 5.1 (Fig. 4d, e). As anticipated, the level of miR-873 expression was greatly reduced in miR-873 KO cells (Fig. 4f); whereas the expression levels of miR-873 targeted genes (e.g. *ARID1B*, *SHANK3*, *ADNP2*, *ANK2* and *CHD8*) were conversely upregulated (Fig. 4g). We note that *ADNP2, CDH8 and ARID1B* have functions related to transcription and chromosome remodelling^39–41^. These data suggest miR-873 may function to suppress neuronal differentiation.

## Discussion

Using whole-exome sequencing, our laboratory has identified many rare SNVs (mutations) in the functional binding regions (seed sequence) of miRNAs in ASD patients, suggesting these miRNAs play important roles in ASD development^5,16^. Among these was a rare SNV mutation (rs777143952: T > A) associated with mature miR-873 gene, which is highly expressed in human brain. We subsequently conducted a pull-down transcriptome analysis to identify miR-873 targets in a human neuroblastoma cell line SH-SY5Y. Among the genes differentially targeted by WT and Mut miR-873 were notable ASD-risk genes *ARID1B, SHANK3* and *NRXN2*. In this study, we experimentally validated differential targeted genes of WT and Mut miR-873 using dual-luciferase assay and qPCR. We examined changes in morphology and the excitability of primary cultured neurons with transfection of WT and Mut miR-873. We also identified a role of miR-873 in repressing neuronal differentiation with CRISPR/Cas9 gene knockout.

Although cultured SH-SY5Y cells do not reflect in vivo conditions of neurons, they express many neural genes^42–44^, hence they were specifically useful as a ‘mechanistic model’ to examine binding affinity and changes in expression of known ASD-risk genes at measurable levels^17^. Autism risk genes, *ARID1B, SHANK3* and *NRXN2* were specially of interst, using a dual-luciferase assay we confirmed the direct targeting of MRE of *SHANK3* by miR-873 albeit, WT and Mut miR-873 mimics similarly dysregulate *Renilla* luciferase. *ARID1B* mutations previously reported in autism cases^45,46^ also cause autistic-like behaviours in mouse models^47^. Same as with *SHANK3*, targeting *ARID1B* by WT and Mut miR-873 reduced *Renilla* chimeric luciferase expression at similar levels. Like *NRXN1*, *NRXN2* has a direct genetic association with ASD^17^, it is a major contributor to synapse development and changes to its expression, as suggested by our results, may affect synaptic homeostasis. Gene transcripts often contain multiple MREs with slight differences in sequence homology and arguably different binding affinity (e.g. *SHANK3*, Supplementary Table 2) including sites found in introns and alternate spliced gene variants that may independently or together affect mRNA dysregulation. All these target sites would need to be exhaustively tested to determine specific molecular differences between WT and Mut miR-873 in vitro, as reflected by changes to gene expression (Fig. 1e).

Transfection of the mutant miR-873 or inhibitor had significant effects on the morphology properties of primary hippocampal neurons assessed by Sholl analysis. MiR-873 transfected primary hippocampal neurons maintain branches and ramifications similar to control neurons, while mutant/inhibitor transfections resulted in less neuronal complexity (Fig. 2). MiR-873 dysregulated key neural genes including *SHANK3* (Fig. 1c–e) leading to observable changes in the development of cultured primary neurons that reflect previously reported results^4^.

Transfection of the mutant or inhibitor miR-873 had significant effects on the functional properties of primary hippocampal neurons assessed electrophysiologically. Membrane potential was significantly depolarised by 7 mV. Importantly, this standing depolarisation has implications for voltage-gated ion channel function. For example, it would be expected to induce ongoing inactivation of the voltage-gated sodium channels responsible for the upstroke and amplitude of the AP. In contrast to expectations, the amplitude of the AP was enhanced as a result of miR-873 transfection (Fig. 3b). Then, using voltage-clamp mode, an increase in the sodium current was observed and this explains the increase in the amplitude of the AP (Fig. 3d). The depolarisation likely reflects effects on ion channel expression. Shanks, Neuroligins and Neurexins are involved in the development of the synapse, both pre- and postsynaptically and this includes cytoskeletal support for various ion channels and transmitter receptors^48^. Overexpression of *NLGN1* results in an increase in spontaneous EPSC frequency in mouse hippocampal neurons^49^ and this supports the interpretation similar to our observation that miR-873 has a suppressor function, albeit decreasing *NLGN3* increased transmitter release in hippocampus and this could not be rescued by *NLGN1*^50^. Clearly, further investigation is required if we are to fully understand this system. Our results support the notion that miR-873 may negatively regulate translation of these proteins and reduction/removal of this miRNA results in enhanced synaptic activity. This effect was abolished by overexpression of Mut miR-873 or inhibitor.

To further explore the biological function of miR-873 on cell development, we genetically disrupted miR-873 using the CRISPR/Cas9 system in human SH-SY5Y cells. Several clones containing INDELs were generated near the cutting site, the resultant miR-873 KO cells were remarkable because they spontaneously differentiated into neuronal-like cells without application of transformation factors (Fig. 4c and Supplementary Fig. 4). We also sequenced putative clones with normal SH-SY5Y cell morphology, confirming they did not contain any mutations flanking the miR-873 gene. Interestingly, clone #2 cells that showed morphological changes contained a single allelic mutation (heterozygous), similar to the genetic profile of the proband associated with this mutation, indicating possible haploinsufficiency in these cells. Among clones that affected cell phenotype, clone #1 was chosen for further study due to a sizeable 12 nt deletion of miR-873 mature sequence (22 nt) (Fig. 4b). The functional knockout status of clone #1 was characterised in increased expression of ASD-risk genes normally targeted by miR-873. Three genes *ADNP2, ARID1B* and *CHD8* are known be involved in transcription control and cell differentiation^39–41^ while *ANK2* has been shown to bind reversibly to the cytoplasmic domain of L1CAM and mediate linkage to the spectrin-actin cytoskeleton related to axon sprouting and neuronal morphology^51^. Quantitative PCR showed >2-folds increase in expression of selected ASD genes in KO compared to control cells, a result that is commensurate with target enrichment levels observed in SH-SY5Y pull-down results^16^ (Fig. 4g). This suggests miR-873 may act as a repressor of cell differentiation similar to other miRNAs. For instance, miR-124 and miR-125b overexpression has been shown to increase neuronal differentiation^6,52^, while miR-663 represses neuronal differentiation^53^ an observation that resonates with our own result. We also observed 21.4% (301) of genes targeted by miR-873^16^ are also targets of miR-663 while only 8.3% (117) genes are shared targets with miR-124 (Supplementary Data 3).

Considering the spatiotemporal functions of miR-873 are yet to be defined, comparing phenotypes in very different cells can be misleading. We note the role miR-873 plays in repressing neuronal-like differentiation and its absence promoting neurite-like outgrowth in neuroblastoma cells is counterintuative with overexpression of miR-873 increasing complexity in primary mouse neurons. Irrespective of conflicting phenotypes miR-873 likely acts as an switch during development. Whether it is ON or OFF will depend on the molecular environment. In neuroblast cells the molecular configuration might be altered or reflect a role tuned to early development, as determined by molecular targets available for interaction. It is also important to reflect on the genomic context of miR-873 being located within an intron of the neural gene *LINGO2*. We are aware of a number of reported copy number variations of *LINGO2* found in autism cases^54,55^ including ones which disrupt miR-873^56,57^. Considering miR-873 may be co-regulated with *LINGO2*, this gene complex warrants further investigation.

Evidently, miRNAs are configured by evolution to control networks of genes whose expression is triggered during a developmental/functional program or through environmental cues. Understanding how these different noncoding regulatory genes work in parallel to shape genetic expression during development and cell differentiation remains a major challenge for cell biology.

Notwithstanding the miR-873-5p variant was inherited from an unaffected parent, we consider how this rare mutation might additively contributed to ASD risk by overlaying other DNA variations to influence genetic liability in this case. The measurable loss of binding and differential expression of several autism risk genes when combined with the mutation load from the mother with recorded BAP^16^ is particularly relevant. Consistent with this idea is the functional disruption of the neurexin-neuroligin axis in this individual—specifically a putative shift in miR-873 binding affinity involving a number of synaptic genes (*NRXN2, NLGN2,-3,-4X, DLG4, DLGAP3,-4* and *SHANK1,-2,-3*) when combined with the loss of function *NRXN1* coding variant^5,16^. Future mouse KO studies whereby combinations of coding and noncoding variants are placed in the same genetic background will directly address this question. Alternately, the CRISPR/Cas9 system could be used to examine combinations of variants in human induced pluripotent stem (iPS) cells.

In summary our results represent the first functional evidence that miR-873 regulates genes associated with changes in neuronal morphology and cell differentiation that builds upon the association with and significance to autism spectrum disorder.

## Supplementary information

Supplementary Information

Data Set 1

Data Set 2

Data Set 3
