# Supplementary material for: Autism-associated miR-873 regulates ARID1B, SHANK3 and NRXN2 involved in neurodevelopment
Source: Transl Psychiatry. 2020 Dec 1;10:418. doi: 10.1038/s41398-020-01106-8 (PMC7708977; doi:10.1038/s41398-020-01106-8)
Supplement: Supplementary file 1 — Supplementary Information [file 41398_2020_1106_MOESM1_ESM.docx]

**Supplementary Information for**

**Autism associated miR-873 regulates *ARID1B, SHANK3* and *NRXN2* involved in neurodevelopment**

Jing Lu^1,*^, Yan Zhu^1^, Sarah Williams^2^, Michelle Watts^3^, Mary A Tonta^1^, Harold A Coleman^1^, Helena C Parkington^1^, Charles Claudianos^4,*^

Correspondence to: CC [Charles.Claudianos@anu.edu.au](mailto:Charles.Claudianos@anu.edu.au) and JL [jing.lu2@monash.edu](mailto:jing.lu2@monash.edu)

**This PDF file includes:**

**Supplementary Figure Legends**

**Supplementary Figures 1-4**

**Supplementary Tables 1-2**

**Supplementary Figure Legends**

**Supplementary Figures 1-3**

**Supplementary Figure 1.** **A heat map showing differentially enrichment of genes pulled-down by wild-type and mutant miR-873 from SH-SY5Y cells (*N* = 4).**

**Supplementary Figure 2. Cytoscape diagram showing the classification of GO_BP terms associated with wild-type (a) and mutant (b) miR-873 gene targets.**

**Supplementary Figure 3. The pulled-down gene targets of wild-type and mutant miR-873 associated with Autism-risk when mapping with SFARI and Autism KB databases.**

Number of Autism-risk genes as collected by SFARI (light blue) and Autism KB (purple) databases from Wild-type (red) and Mutant (blue) miR-873 pulled-down gene targets.

**Supplementary Figure 4. Neurite outgrowth and neuronal-like differentiation of SH-SY5Y cells after miR-873 knockout with CRISPR/Cas9.** Left panel shows morphology of SH-SY5Y cells, and right panel shows morphology of miR-873 knockout SH-SY5Y cells.

**Supplementary Tables 1-2**

**Supplementary Table 1. Primer list.**

**Supplementary Table 2. Wild-type and mutant miR-873 MREs of *SHANK3* in human and mouse by MIRANDA prediction.**

**Supplementary Data 1-3**

**Supplementary Data 1.** **Total wild-type and mutant miR-873 pulled down genes**. Analysis of the total wild-type and mutant miR-873 pulled down genes, with *P* ≤ 0.01, fold change ≥ 2.

**Supplementary Data 2. Enriched GO_BP terms of wild-type and mutant miR-873 pulled-down genes respectively**.

**Supplementary Data 3. miR-873 pulled-down targets sharing with miR-124, miR-125 and miR-663.**

**Supplementary Figures**

**Supplementary Figure 1.**

**
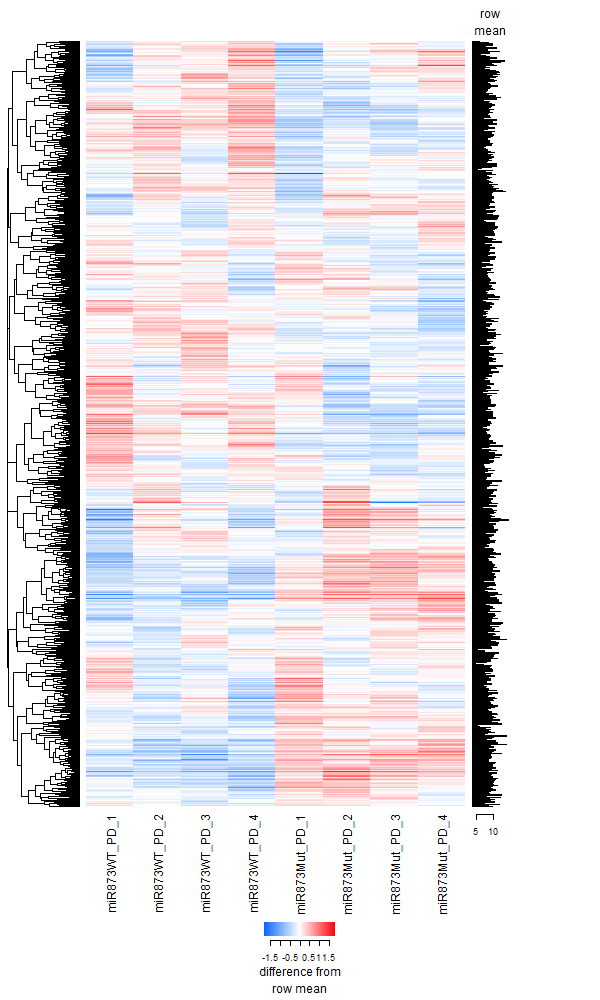
**

**Supplementary Figure 2.**

a

b

**Supplementary Figure 3.**

Mut

WT

746

651

322

15

7

41

Autism KB

SFARI

13

24

106

SFARI

Autism KB

25

17

94

SFARI

Autism KB

**
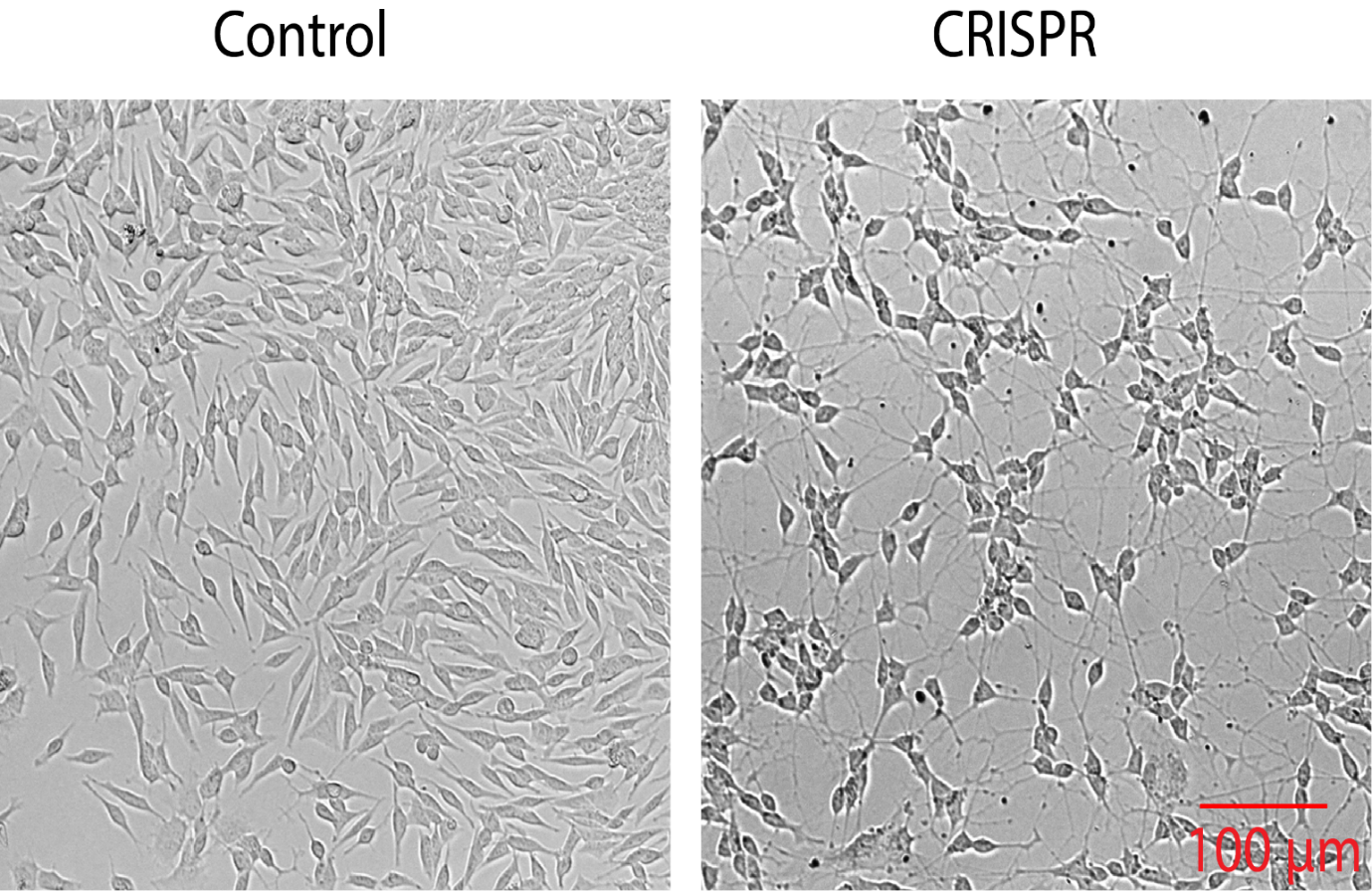
Supplementary Figure 4.**

**Supplementary Tables**

**Supplementary Table 1.**

| **Primers for Dual-luciferase assay** | |
| --- | --- |
| **Primer name** | **Sequence (5' -> 3')** |
| XhoI cut site_For | CAGTGACTCTCGAGCAG |
| XhoI cut site_Rev | CTGCTCGAGAGTCACTG |
| Not I cut site_For | GACGCGGCCGCCAGTGACT |
| Not I cut site_Rev | AGTCACTGGCGGCCGCGTC |
| miR873-positive control_For | CAGTGACTCTCGAGCAGAGGAGACTCACAAGTTCCTGCGACGCGGCCGCCAGTGACT |
| miR873-positive control_Rev | AGTCACTGGCGGCCGCGTCGCAGGAACTTGTGAGTCTCCTCTGCTCGAGAGTCACTG |
| NLGN2_For | CAGTGACTCTCGAGCAGACCAGACCACAGAGGATGGA |
| NLGN2_Rev | AGTCACTGGCGGCCGCGTCACACATCCACATGCAAGAACC |
| SHANK3_For | CAGTGACTCTCGAGCAGGCAGCCATCTTCCTCACAGG |
| SHANK3_Rev | AGTCACTGGCGGCCGCGTCCTCTCACAGGTACACCCCAC |
| SYNGAP1_For | CAGTGACTCTCGAGCAGTGCAGATTACGGAGAACGGC |
| SYNGAP1_Rev | AGTCACTGGCGGCCGCGTCCTCCCTCCTGGAAATGGTGC |
| ARID1B_For | CAGTGACTCTCGAGCAG TGTCCTGAACTCTCTGGTTGC |
| ARID1B_Rev | AGTCACTGGCGGCCGCGTC ATGGGGCAGAGGAGCAAAGA |
| SHANK3 MRE_For | CAGTGACTCTCGAGCAG CTTGGACCCCAAAGTCTCCTGT GACGCGGCCGCCAGTGACT |
| SHANK3 MRE_Rev | AGTCACTGGCGGCCGCGTCACAGGAGACTTTGGGGTCCAAGCTGCTCGAGAGTCACTG |
| SHANK3 MRE_Mut_For | CAGTGACTCTCGAGCAG CTTGGACCCCAAAGTCTAAGGT GACGCGGCCGCCAGTGACT |
| SHANK3 MRE_Mut_Rev | AGTCACTGGCGGCCGCGTCACCTTAGACTTTGGGGTCCAAG CTGCTCGAGAGTCACTG |
| **Primers for qPCR** | |
| **Primer name** | **Sequence (5' -> 3')** |
| SHANK3-qFor | GTCCTGCTCTTCCGTGGAG |
| SHANK3-qRev | TGGGTCTTGATAACCTCTGCAA |
| NLGN2-qFor | TGGTTCACCGACAACTTGGAG |
| NLGN2-qRev | GCACGTAGAGGTTGAGGTACAG |
| ARID1B-qFor | CAAGGGGATCAGAGCAACCC |
| ARID1B-qRev | CTACCTGGGATACTTGCAGGA |
| SYNGAP1-qFor | CCTTCAGAGATGTACGGGGAC |
| SYNGAP1-qRev | GTTCCAACCAGGACGATCATAC |
| NRXN2-qFor | CAGCACGAGGATGGATCGC |
| NRXN2-qRev | GCCCACGTTAAAGATCACCCC |
| DLG4-qFor | TCGGTGACGACCCATCCAT |
| DLG4-qRev | GCACGTCCACTTCATTTACAAAC |
| DLGAP4-qFor | GGATGCCAATGACTCAAGCTG |
| DLGAP4-qRev | TTAGGAACCAGTAGCCGTCTC |
| GAPDH-qFor | CAAGAAGGTGGTGAAGCAGGC |
| GAPDH-qRev | GGCATCGAAGGTGGAAGAGTG |
| CHD8-qFor | TACAGCAGGCTCAGATAATGGG |
| CHD8-qRev | TTGCTTCTTCTGGTGTTCCAAT |
| ANK2-qFor | ACCTGCAATCAGAATGGACTCA |
| ANK2-qRev | TGCAATGTGAAGAGCGGTATT |
| ADNP2-qFor | AGGACCTTAAAGGCTTTGATCCA |
| ADNP2-qRev | ACAACAGTATGGCTTTGTTCGAT |
| U6 snRNA-qFor | CGCTTCGGCAGCACATATAC |
| U6 snRNA-qRev | AAAATATGGAACGCTTCACGA |
| **Primers for qPCR detection of miR-873** | |
| miR-873-StemRT | GTCGTATCCAGTGCAGGGTCCGAGGTATTCGCACTGGATACGACAGGAGA |
| UNI-qPCR-StemRT | CCAGTGCAGGGTCCGAGGTA |
| SL-has-miR-873-qF | CACGCAGCAGGAACTTGTGAG |
| **Primers for CRISPR/Cas editing** | |
| gRNA-miR-873-For | CACCGTCTCCTATTGAAAATGAAC |
| gRNA-miR-873-Rev | AAACGTTCATTTTCAATAGGAGAC |
| miR-873-Seq_For | CTTAAGGGATGCTCAATCAATAT |
| miR-873-Seq_Rev | GTGGGATTCAACACCTCAAGTA |
| Primers for off-target detection | |
| NM_015378 _For | TCAGTTTGCACTGGGTTTCAA |
| NM_015378 _Rev | AGATATCGCCACCTTGGGTT |
| NM_001200001_For | AGGTATATTCCACTTTCAGGGTTT |
| NM_001200001_Rev | CAGTAATGCGTGTTGCCAGC |
| NM_152745_For | TTTCTCTTCTGTTTTCAGGTCACA |
| NM_152745_Rev | CGTTTTAACAATGGGCCTTCTCT |
| NM_173569_For | TTGGCTTTGGAACGGACACA |
| NM_173569_Rev | AACTCCAACTGGACTACTCTT |
| NM_004760_For | TGTTTTTGTTTAATTCTAGGGGGAA |
| NM_004760_Rev | AATAAGAGGAACACCTGAAGATGA |

**Supplementary Table 2.**

|  | Sequence match | Energy (kcal/mol) |
| --- | --- | --- |
| miR-873_WT  h-*SHANK3*_MRE1 | 3' tcctcTGAGTGTTCAAGGACg 5'  \|\|\|\|\|\|\|\|\|\|\|:\|\|\|  5' acgtcACTCACAAGTTTCTGt 3' | -25.41 |
| miR-873_WT  h-*SHANK3*_MRE2 | 3' tcctctgagTGTTCAAGGACg 5'  :\|\|\|\|\|\|\|\|\|\|  5' gccgcgccgGCAAGTTCCTGg 3' | -19.15 |
| miR-873_WT  h-*SHANK3*_MRE3 | 3' tcctcTGA-GTG-TTCAAGGACg 5'  :\|\| \|\|\| : \|\|\|\|\|\|\|  5' gcgccGCTCCACTGTGTTCCTGt 3' | -17.41 |
| miR-873_Mut  h-*SHANK3*_MRE4 | 3' tcCTCTGAGTGTTCAAGGTcg 5'  \|:\|:\| \| \|::\|\|\|\|\|\|  5' ctGGGGCCCTCGGGTTCCAcc 3' | -21.42 |
| miR-873_WT  m-*SHANK3*_MRE1 | 3' tcctcTGAGTGTTCAAGGACg 5'  \|\|\|\|\|\|\|\|\|\|\|:\|\|\|  5' acgtcACTCACAAGTTTCTGt 3' | -25.41 |
| miR-873_WT  m-*SHANK3*_MRE2 | 3' tcctctgagTGTTCAAGGACg 5'  :\|\|\|\|\|\|\|\|\|\|  5' gtcgcgccgGCAAGTTCCTGg 3' | -18.32 |
| miR-873_WT  m-*SHANK3*_MRE3 | 3' tcctctGAGTGTTCAAGGACg 5'  \|\|\| \| \|\|\|\|\|\|\|  5' cactccCTCTCCTGTTCCTGc 3' | -19.43 |
| miR-873_WT  m-*SHANK3*_MRE4 | 3' tcctctGAGTGTTCAAGGACg 5'  \|\|\|\|\|\| \| \| \|\|\|\|\|\|  5' caaAGACTCCCCAATTCCTGc 3' | -21.92 |
| miR-873_Mut  m-SHANK3 | 3' tcCTCTGAGTGTTCAAGG**T**cg 5'  No hit | 0 |
